# PLR-TV: patch-based low rank with spatio-temporal total variation constraints for ungated myocardial perfusion CMR

**DOI:** 10.1186/1532-429X-16-S1-W22

**Published:** 2014-01-16

**Authors:** Ganesh Adluru, Edward V DiBella

**Affiliations:** 1 Radiology, University of Utah, Salt lake city, Utah, USA

## Background

Ungated myocardial perfusion is a promising alternative for simplifying CMR protocols [1]. Spatio-temporal total variation (TV) constrained reconstruction with radial undersampling was used in a pilot study [2]. And TV constraints combined with a low-rank constraint have shown improvement in some cases for gated perfusion imaging [3]. However, the temporal total-variation constraint may not effectively resolve undersampling artifacts in ungated studies with significant cardiac and respiratory inter-frame motion. A patch-based low-rank method can more effectively exploit redundancies in a dynamic dataset in the form of spatio-temporal patches rather than one large rectangular matrix with an entire image per column. The patch-based method (also termed locally low rank or blockwise low rank) was recently shown to improve upon the standard low-rank constraint in cardiac cine and perfusion imaging [4,5]. Here we adapt a patch-based low-rank method for ungated myocardial perfusion imaging and use it in conjunction with TV constraints.

## Methods

**Figure 1:**
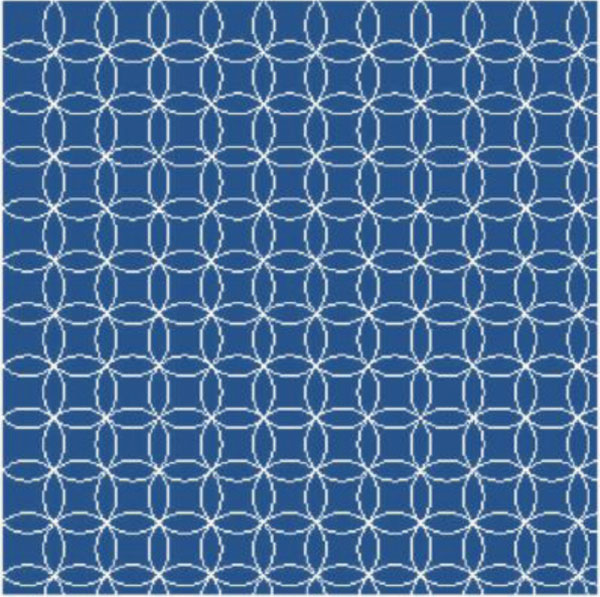
**Image showing approximately circular patches that are overlapping on a Cartesian grid for PLR constraint**.

Ungated perfusion data was acquired for eight patients on a Siemens 3T Verio scanner using a saturation recovery sequence [2] at rest and at pharmacologically induced stress with TR = 2.2 msec, TE = 1.2 msec, FOV = 280 mm2, matrix size = 144 × 20. Five slices were acquired in 250 msec. Patch-based low-rank with spatio-temporal TV constraints was applied in a Projection Onto Convex Sets (POCS) based alternating framework [6,7] while enforcing data fidelity. We used overlapping patches that are approximate circles on Cartesian grid as shown in Figure 1 with a radius of 8 pixels. The overall amount of spatial overlap in the image was < 60%.

## Results

**Figure 2:**
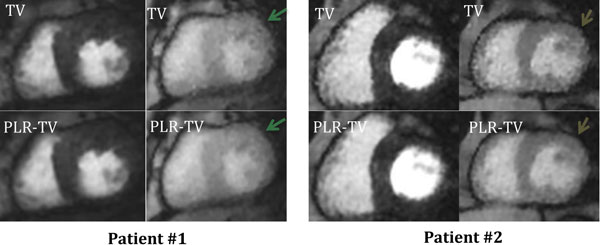
**Comparison of reconstructed image quality for ungated radial perfusion imaging for two patient datasets**. Near systolic frames pre and post contrast are shown. Images labeled TV are reconstructions with spatio-temporal TV constraints and those labeled with PLR-TV are corresponding images with patch-based low ran and spatio-temporal TV constraints.

Figure 2 compares the results of the PLR-TV with TV reconstructions. PLR-TV images have less pixelation artifacts than the TV reconstructions and have improved delineation of papillary muscles and myocardial boundaries especially in post-contrast frames.

## Conclusions

PLR-TV is a promising approach for reconstructing undersampled radial ungated data. While the PLR constraint exploits the low rank property effectively in the ungated images, TV constraints can help preserve any contrast loss from the PLR, making the hybrid method more effective than either constraint alone in the presence of motion. Here we chose overlapping circular patches over time (so that the Casorati matrix is almost square), although arbitrary shaped patches can be chosen depending on underlying geometrical structures. While adding PLR constraints increases the reconstruction time, we limited the amount of overlapping of the circular patches so as to cover all of the pixels in the image for the PLR constraint. Randomized patch updates and the use of TV reconstructions as initial estimates for the final PLR-TV reconstructions can reduce the computational cost.

## Funding

RO1 HL113224.
